# Unpacking the link between flexible work arrangements and job stress: The mediating role of Job satisfaction among highly educated employees

**DOI:** 10.1371/journal.pone.0335279

**Published:** 2025-10-23

**Authors:** Dimitrije Gašić, Nemanja Berber, Nikola Ćurčić, Mladen Čudanov, Tibor Zsigmond, Darko Tomaš

**Affiliations:** 1 University of Novi Sad, Faculty of Economics in Subotica, Subotica, Serbia; 2 University of Belgrade, Institute for Multidisciplinary Research, Belgrade, Serbia; 3 University of Belgrade, Faculty of Organizational Sciences, Belgrade, Serbia; 4 J. Selye University, Faculty of Economics and Informatics, Komárno, Slovakia; 5 University of Banja Luka, Faculty of Economics, Banja Luka, Republic of Srpska, Bosnia and Herzegovina; Shenzhen University, CHINA

## Abstract

The main objective of this research was to investigate the mediating role of Job satisfaction in the relationship between flexible working arrangements (FWAs) and Job stress. The main research question is: what is the nature of the effect of flexible working arrangements on Job stress and Job satisfaction, the effect of Job satisfaction on Job stress and the role of Job satisfaction as mediation in the relationship between FWAs and Job stress? The methodology consists of theoretical and empirical parts. The empirical research was performed on a sample of 448 highly educated employees who operate in Serbia. Sample collection lasted from April to July 2024 via G-Forms electronic questionnaire. The proposed relationships were tested by applying PLS-SEM method via SmartPLS software. The main findings of the research are that there are positive effects of Flexible work arrangements and Job satisfaction on Job stress, and Job satisfaction has an indirect effect on the relationship between Flexible work arrangements and Job stress. The results indicated a partial mediation; a noticeable reduction of job stress was observed among employees who are sati.sfied with their jobs because of the implementation of flexible work arrangements.

## 1. Introduction

Flexible work arrangements are becoming an increasingly important aspect of the modern work environment, especially considering the changes brought about by globalization, the development of digitalization, and various economic, health (Covid-19), political, and other crises [1,2]. Legesse Bekele and Mohammed [3] emphasize that the rapid changes in the characteristics of the global workforce, as well as trends in talent management, are forcing companies to develop innovative strategies for attracting, motivating, and retaining employees. These strategies must become part of management and corporate culture, and personnel at all levels should be involved in their implementation. Pradère and Taylor [4] emphasize that the role of leaders is crucial; they need to lead and embrace new tasks to create a supportive work environment that will mitigate increased stress levels, prevent demotivation, and improve working relationships. As more employers adopt this approach to meet the needs of their employees better, it has become essential to examine the impact of such methodologies on key work dimensions, such as job satisfaction and job stress. Since these are two critical dimensions of work that have a direct impact on work efficiency and the overall experience of employees in the workplace, exploring how flexible work arrangements can contribute to increased job satisfaction and reduced stress levels can assist human resource managers in achieving an optimal organizational climate, leading to improved business outcomes. Job satisfaction and stress are two important factors affecting work efficiency and employee well-being.

Understanding how FWAs can enhance job satisfaction and reduce stress can help organizations optimize their work environment, resulting in better business performance and increased competitiveness in the market. Additionally, the mediating role of job satisfaction in the relationship between FWAs and job stress offers further insight into how these changes impact employees’ emotional and psychological state. Researching these relationships is significant for theoretical understanding and practical implementation in organizations striving to establish a healthy and productive work environment. Previous studies indicate that there is no universal and unambiguous approach to the implementation of FWAs, as their application largely depends on the institutional framework, the socio-economic context of a given country, and the organizational culture itself [3]. This heterogeneity suggests that FWAs cannot be uniformly applied across different countries, which additionally creates a research gap in understanding specific national and regional factors that shape the effects of these arrangements on job satisfaction and job stress. It is therefore necessary to pay special attention to examining this issue in transitional and post-socialist economies such as Serbia, where FWAs are still not sufficiently developed or institutionally embedded.

Therefore, this study aims to provide relevant data and recommendations that can aid in shaping future work policies, particularly in the context of the growing need to balance professional obligations and employees’ personal lives in development country of Serbia. Creating a healthy organizational culture can positively impact employees, as they feel they are creating value through their work, with the role of managerial support being of great importance. Garai-Fodor and Jäckel [5] emphasize that a healthy organizational culture can be based on openness and trust. Every organization should create an adequate organizational culture with the goal of becoming a desirable company to reduce job stress, turnover intentions etc., Habib et al. [6] emphasized that a company must satisfy its numerous stakeholders (including employee satisfaction) to prevent the formation of negative perceptions about its business operations. Piwowar-Sulej et al. [7] emphasizes that the mission of HRM is to support the organization in achieving its goals through the implementation of HR strategies that will positively influence the promotion of employee development, better relationships between employees, and so on. Adequate HRM is focused on the performance of employees, teams, or the entire organization [8]. Therefore, it is essential for managers at all levels to develop adequate strategies and positively impact the reduction of stress and turnover intentions, which are some of the main organizational challenges in today’s post-COVID business environment.

The paper is structured into three main sections. The first section focuses on the theoretical background of the research, where the authors reviewed professional literature on the topics of Flexible work arrangements, Job satisfaction, and Job stress. Following this, the authors examined previous theoretical and empirical findings on the relationships between FWAs and Job stress, FWAs and Job satisfaction, Job satisfaction and Job stress, as well as the mediation role of Job satisfaction between FWAs and Job stress, and based on this, they created the conceptual framework for the research. This conceptual framework has not been explored in Serbia or beyond, making it a significant scientific contribution to the research field. The next section is dedicated to the research methodology, where the authors provide an overview and explanation of the questionnaire used for data collection and sampling procedures. Then, the research sample on which the relationships were examined is examined in the subsequent section. The third main section focuses on analysing and discussing the obtained data. The authors applied the PLS-SEM method using the SmartPLS statistical software for data processing. In this section, the research hypotheses were tested based on previous theoretical and empirical findings by other authors. The aim was to determine whether the hypotheses were confirmed within the sample formed in the territory of the Republic of Serbia. The following section pertains to the concluding considerations, where the authors summarized the research and provided recommendations to companies on how they can positively influence increased job satisfaction and reduce employee job stress.

## 2. Theoretical background

The second part focuses on the theoretical explanation of the variables to be studied: Flexible work arrangements, Job satisfaction, and Job stress. This is followed by a review of existing theoretical and empirical findings on the relationships between FWAs, Job satisfaction, and Job stress, as well as the mediating role of Job satisfaction in the relationship between Flexible work arrangements and Job stress. Based on the review of previous research, the authors have developed a conceptual framework for the study, presented at the end of this section.

### 2.1. Flexible work arrangements

Flexible work arrangements represent a set of work practices that provide employees with greater flexibility and freedom regarding the location and timing of their work activities [9]. These arrangements deviate from the traditional work model with fixed hours and location, allowing employees to align their work obligations with personal needs and lifestyle [10]. They represent a modern way of working that is becoming increasingly prevalent worldwide due to its advantages. The most significant theoretical foundation of FWAs is the social exchange theory (SET), which suggests that individuals engage in social relationships and interactions with others based on perceived exchanges of rewards and costs. In FWAs, mutual benefits are expected for both employees and the organization. According to SET, employees perceive FWAs as a form of reward that can improve work-life balance (WLB), create overall well-being, and positively influence attitudes and behaviour [11,12]. Employees assess the rewards they receive for their work, considering whether they are proportional to the costs and inputs they bear. For instance, an employee might perceive injustice if required to work longer hours or feel isolated due to remote work without adequate compensation, leading to dissatisfaction and reduced employee commitment [13].

Additionally, they need to provide the necessary resources, training, and effective communication channels to help employees better adapt to the implementation of FWAs. Kelliher and De Menezes [14 pp 4–6] emphasize that companies can more effectively manage their human resources through job redesign and flexible work arrangements. Through an exploration of different types of FWAs, the authors identified a classification of 12 types based on the research of [15] Stavrou (2005) and the Cranet international research methodology [16,17]. These FWAs include weekend work, shift work, overtime, flexi-time, home-based work, remote work, compressed working week, job sharing, part-time work, fixed-term contracts, temporary work, and annual hours contracts. These arrangements provide better work-life balance [18,19], can increase job satisfaction [20–23] and productivity [24,25], and reduce stress levels among employees [26,27]. However, the effectiveness of these arrangements depends on the nature of the work, organizational culture, and individual employee preferences.

### 2.2. Job satisfaction

As an academic concept, job satisfaction has garnered significant attention from experts in management, practical business, and social psychology in recent years. Weiss [28] citing Locke [29] job satisfaction is defined as “a pleasant emotional state resulting from the appraisal of one’s job as achieving or facilitating one’s work values.” Job satisfaction refers to the level of fulfillment an individual experiences in their job or occupation, influenced by various factors such as the nature of the job itself, the work environment, compensation system, relationships with colleagues and supervisors, opportunities for career growth and advancement, and alignment of personal values with organizational goals. It is one of the most frequently used variables in organizational behavior and can be defined as an individual’s cognitive, affective, and behavioral response to their job [30]. The cognitive dimension focuses on an individual’s evaluation and perception of their job, which includes beliefs, thoughts, and perceptions about various aspects of their work, such as tasks, workload, compensation, and opportunities for growth and development. Boohene and Williams [31] explained that the cognitive dimension encompasses beliefs and opinions about change depending on positive and/or negative evaluations. Erwin and Garman [32] emphasize that the cognitive dimension reflects how employees think about and view changes, such as the advantages, disadvantages, and values of implementing those changes. The affective dimension relates to employees’ emotional or affective response to their job and encompasses positive or negative feelings and emotions associated with work experiences. Happiness, fulfilment, and enthusiasm are experienced by employees who are affectively satisfied with their job, while stress, frustration, and boredom occur in employees with low affective job satisfaction. Employees’ perceptions, assumptions, and decisions to adjust their behaviour are emotionally driven [33]. The behavioural dimension includes observable behaviors that individuals exhibit in response to their level of job satisfaction and encompasses employees’ reactions to changes and their intentions regarding how to respond to implemented changes [34]. Finally, changes in job satisfaction could have numerous positive, but also negative consequences, both for the individual and for the organization [35].

### 2.3. Job stress

Michigan group’s perspective [36], stress can be viewed as individuals’ reactions to seemingly overwhelming work environment characteristics [27]. This indicates a poor alignment between individuals’ capabilities and their work environment, where either excessive demand are placed on individuals or they are not fully equipped to handle a particular work situation [37]. Lamontagne et al. [38] emphasize that interventions to alleviate job stress have multiplied rapidly over the last two decades, due to the increasing recognition and acceptance of the adverse impacts of job stress on individuals and organizations.

Gupta and Beehr [39] emphasize that stress is extremely aversive for most employees and creates a harmful situation in the work environment. Various examples from several major psychological theories (e.g., field theory, balance theory, and reinforcement theory) can be used to demonstrate that individuals tend to avoid unpleasant situations. If the work organization is unpleasant, there is a high likelihood that the individual will try to avoid it by being late, being absent, or even quitting.

An indirect relationship between stress and withdrawal based on the mediating effects of job dissatisfaction is possible, and studies by [40,41], and [42] have shown that stress has a positive correlation with job dissatisfaction. Meanwhile, the study by Berber et al. [43] showed that job satisfaction mediates the relationship between flexible work arrangements and employees’ intention to leave, indicating that if employees are satisfied with their jobs and can use FWAs, they will be less likely to want to leave their job. Accordingly, it can be assumed that employees who are satisfied with their jobs will experience lower level of job stress. Haley and Miller [44] emphasize that the workplace is the primary cause of stress related to employees’ health. Specifically, the Center for Disease Control notes that “problems at work are more strongly associated with health problems than any other source of stress in life—even more so than financial or family problems.” They also highlight that up to 40% of workers have a very or extremely stressful job. These data imply that workplace stress effectively functions as a widespread health risk. It is essential for companies to make necessary changes in their business practices and behavior patterns to reduce stress and, consequently, address sleep-related issues. Sato, Kuroda and Owan [45] emphasize that working at night negatively affects employees’ health. Consequently, according to the Labor Law of the Republic of Serbia [46], Article 108, employees have the right to an increased salary, as determined by the general act and employment contract, for night work if such work is not accounted for when determining the base salary, by at least 26% of the base amount.

### 2.4. Relationship between Flexible work arrangements and Job stress

Russell, O’Connell and McGinnity [47] highlight that the recent rapid economic growth in Ireland has led to an increase in the number of employed women, which has, in turn, led to a rise in the proportion of dual-earner families. Consequently, these changes have brought the issue of adequately WLB to the forefront. FWAs in organisations have been identified as one of the important WLB and employees commitments. The results indicate that part-time and flexi-time reduce work pressure and work-life conflict, while home-based work is associated with higher levels of both. The authors emphasize the need to distinguish between FWAs to uncover their potential for reducing work pressure and work-life conflict. Parker and DeCotiis [48] point out that organizational pressure negatively contributes to increased job stress.

Chandola et al. [27] found that employed men and women who had the option to work reduced hours had lower levels of allostatic load, defined as the cumulative burden of chronic stressors and personal life events. Among women caring for two or more children under the age of 15, there was a difference of almost one unit in the allostatic load index compared to those who did not work reduced hours. They highlight that reducing working hours for women could positively impact the reduction of chronic stress levels.

Mache, Servaty and Harth [49] found in a sample of employees from a large technology company that the transition to open workspaces led to significant changes in working conditions. Mental demands decreased, while workload increased. Communication, team collaboration, and job autonomy improved, and levels of occupational stress significantly decreased over time. Results from regression analysis indicated a significant association between FWAs, job resources, and reduced occupational stress. These findings contribute to understanding the effects of flexible work arrangements on employee well-being and performance in open workspaces.

Grzywacz, Carlson and Shulkin [50] found in a sample of 19,704 employees from various industries that stress, and burnout levels were lower among those involved in all types of formal flexible arrangements. Results indicated that 30–50% of the observed differences between workers using flexitime and those not engaged in formal arrangements were explained by perceived flexibility. These results provide clear support for advocates’ calls for employers to expand FWAs, particularly flexitime.

Based on the previous theoretical and empirical knowledge of the authors, the first research hypothesis is proposed:

**Hypothesis 1 (H1).** Flexible work arrangements are negatively related to Job stress.

### 2.5. Relationship between Flexible work arrangements and Job satisfaction

The results of Xiang et al. [51] indicate that the use of flexi time is positively associated with specific indicators of job satisfaction, with the association being stronger among mothers who had access to formal FWAs. The results suggest that while formality is important, it can also intensify the tension between the benefits and penalties associated with flexi time.

Jung and Yoon [52] found, based on a sample of 277 employees from Generations X, Y, and Z working in luxury hotels in South Korea, that the possibility of applying FWAs is positively related to job satisfaction. The results indicate that Generation Z places significant importance on job flexibility and that luxury hotels should develop flexible policies and adjust their organizational climate if they wish to influence j satisfaction, work engagement, and commitment positively.

Berber et al. [43] examined a sample of 219 employees in Serbia using PLS-SEM, and identified positive effects of FWAs and job satisfaction on employees’ turnover intentions. The study found indirect effects of FWAs on turnover intentions through job satisfaction. FWAs can contribute to increased job satisfaction, which in turn helps to reduce turnover intention. Job satisfaction mediates this relationship, and employees who are offered the possibility of FWAs may experience lower levels of turnover intention when they are satisfied with their jobs.

Arntz, Yahmed and Berlingieri [53] found that employees without children work an additional hour of unpaid overtime per week and report higher job satisfaction by using the “home-based work.”

Palumbo et al. [54] examined a sample of 16,473 employees, of which 54.4% were men and the remainder women, with an average age of 40 years. They found that remote work fosters improved work motivation, enhancing job satisfaction. Job satisfaction and increased work motivation nurture a positive perception of employees regarding the balance between work-life. Similarly, Taboroši et al. [55] found that teleworkers showed higher level of job satisfaction that conventionally employed persons.

Based on the previous theoretical and empirical knowledge of the authors, the first research hypothesis is proposed:

**Hypothesis 2 (H2).** Flexible work arrangements are positively related to Job satisfaction.

### 2.6. Relationship between Job satisfaction and Job stress

Job satisfaction is often linked to a low level of stress at work. When employees feel that their efforts are recognized and have a good work-life balance, it can significantly reduce stress levels. Additionally, management support, clear communication, and opportunities for professional development can contribute to greater satisfaction and reduced stress.

Lelis et al. [56] found, based on a sample of 114 managers and using Spearman’s correlation, that stress has a negative correlation and statistical significance with five dimensions of job satisfaction, indicating that stress negatively affects job satisfaction. Masihabadi et al. [57] measured the effects of job stress on job satisfaction, organizational commitment, and employee performance among auditors in audit institutions in Tehran and Mashhad. The results indicated that job stress has a negative impact on job satisfaction and organizational commitment. Job stress also negatively affected job satisfaction through organizational commitment. An, Zhang and Lee [58] investigated the relationship between job stress and job satisfaction among construction project managers. Pearson’s correlation results indicate that field managers who were satisfied with their job reported lower levels of job stress. The findings can be used to manage construction site managers effectively and improve their job performance in the field. Marzabadi and Tarkhorani [59] highlight that stress is one of the main contributors to medical illnesses among employees and that numerous studies have shown that many individuals experience stress at work due to various circumstances. The authors’ findings help understand the factors contributing to an adequate work environment and suggest ways to reduce stress and increase job satisfaction. According to Chandraiah et al. [60] various studies have shown a correlation between occupational stress and job satisfaction. These studies have proven that the stress level experienced by workers affects their job satisfaction. This means that workers who experience a high stress level will be less satisfied with their work [61].

Based on the previous theoretical and empirical knowledge of the authors, the first research hypothesis is proposed:

**Hypothesis 3 (H3).** Job satisfaction are negatively related to Job stress.

### 2.7. Mediating role of Job satisfaction in the relationship between Flexible work arrangements and Job stress

A review of theoretical and empirical knowledge on the mediating role of job satisfaction in the relationship between FWAs and job stress has revealed a scarcity of research. The mediating role of job satisfaction in this relationship refers to how job satisfaction can explain the impact that FWAs have on job stress. When employees have the opportunity for more flexible work, it often increases their job satisfaction, which in turn can reduce the level of job stress. In other words, flexible work arrangements may directly reduce job stress but indirectly contribute to stress reduction by increasing job satisfaction.

Ghimire, Dahal and Rai [62] highlight that with the demand for greater exposure and better job results, there has been an increase in pressures that affect higher levels of stress among employees, as well as productivity, dissatisfaction, and turnover intention of employees. Burke and Cooper [63] emphasize that, in this context, flexibility in work operations would allow employees to better align their work hours according to their preferences.

Kelliher and Anderson [64] investigated employees’ experiences using FWAs such as home-based work, weekend work, and part-time job. The results were consistent with numerous existing studies, showing that employees who utilized FWAs reported higher levels of job satisfaction [65–67] and organizational commitment [68,69] compared to their counterparts who did not use these FWAs. Additionally, lower stress levels were reported in the case of employees working reduced hours [70,71].

Based on the previous theoretical and empirical knowledge of the authors, the first research hypothesis is proposed:

**Hypothesis 4 (H4).** Job satisfaction has a positive mediation effect in the relationship between Flexible work arrangements and Job stress.

**Fig 1:**
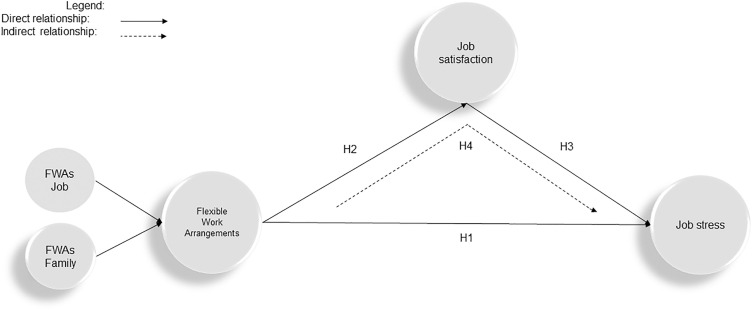
Contextual framework. Source: The authors’ research.

Based on theoretical and empirical knowledge about the mentioned relationships, a conceptual framework for the research has been developed (Fig 1).

## 3. Methodology

The third section of the paper is focused on a detailed explanation of the questionnaires used for data collection, based on which the data analysis will be conducted. This section provides a precise description of the sampling procedure and the way the data were organized. The sample is presented in a tabular form and further described to better understand its structure and relevance for subsequent analysis.

### 3.1. The questionnaire

The questionnaire is composed of four segments. The first part includes control questions (gender, age, education level, etc.). The second part examines the independent variable “Flexible work arrangements,” consisting of 11 statements adapted from Albion [72]. Factor analysis categorized these statements into two groups: the first group consists of job-related statements (“FWAs - Job”: FWAs1, FWAs5, FWAs6, and FWAs7), while the second group consists of family-related statements (“FWAs - Family”: FWAs2R, FWAs3R, FWAs4R, FWAs8R, FWAs9R, FWAs10R, and FWAs11). The third part of the questionnaire measures the dependent variable “Job Satisfaction,” which acts as a mediator in this model and consists of 5 statements adapted from Morgeson and Humphrey [73]. The fourth and final part measures the dependent variable, “Job Stress,” composed of 6 statements adapted from Lait and Wallace [74]. The authors created an electronic version of the questionnaire using the “Google Forms” platform, enabling respondents to complete it at any time and location using their devices. This significantly facilitated data coding and preparation for analysis.

### 3.2. Sampling procedure

Based on the defined subject and objectives, the research includes employees operating in the territory of the Republic of Serbia. Data collection through Google Forms was conducted from April to July 2024. A total of 448 employees completed the questionnaire, all of whom use flexible work arrangements. Before completing the questionnaire, all respondents were informed of the purpose of the research, and it was explained to them in the preamble of the questionnaire that completion was voluntary and anonymous and that the data would be used for research purposes only. If they decided to complete the questionnaire, they could leave the research at any time without any consequences. It was also emphasized that by completing the questionnaire they were giving their consent to participate.

Data obtained from the website of the Statistical Office of Serbia indicates that the total number of employees in the second quarter of 2024 was 2,367,170 [75]. According to the size of the target population in the research for the year 2024, and by the sample size formula developed by Cochran (1997), it is concluded that for the most applied significance level of 5% and a confidence interval of 95%, a sample in the research can be considered of at least 385 employees in Serbia. Based on the above, it can be concluded that this criterion has been met. Below is a tabular of the sample on which the research was conducted within point 4.

### 3.3. The sample

The sample consists of 448 employees from the territory of Serbia who use various forms of FWAs. Data collection was carried out from April to July 2024. The authors focused on highly educated, young employees, as they are the ones who most frequently use FWAs. Table 1. refers to sample characteristics presentation.

**Table 1:** Sample characteristics (∑ 448).

Category	Subcategory	%	Number of Employees (N)
**Gender**	Male	44%	197
	Female	56%	251
**Age**	Less than 25 years	18%	81
	25 - 34 years	43%	193
	35 - 44 years	39%	174
**Level of education**	Bachelor	56%	251
	Master	35%	157
	PhD	9%	40
**Sector**	Private	78%	350
	Public	22%	98
**Number of jobs**	1 job	60%	269
	2 jobs	24%	108
	More than 2 jobs	16%	72
**Position in company**	Manager	34%	152
	Professional worker	66%	296

Source: The authors’ research.

Based on the presented table, it can be concluded that female (56%; N = 251) make up most of the sample, while the rest are male (44%; N = 197). When comparing the age structure, it is noticeable that the sample consists of a younger employee, with the largest number between 25–34 years old (43%; N = 193), followed by those 35–44 years (39%; N = 174), while the smallest number are under 25 years old (18%; N = 81). The sample consists of highly educated employees, which corresponds to the research topic, as this type of work arrangement is more common among highly educated employees in higher hierarchical positions. Based of the data obtained, the largest number of them have bachelor’s degree (56%; N = 251), followed by a Master’s degree (35%; N = 157), and a Ph.D. (9%; N = 40). Regarding their position in the company, the majority work as professional workers (66%; N = 296), while the rest managers (34%; N = 152). Regarding sector affiliation, most are employed in the private sector (78%; N = 350), while the rest work in the public sector (22%; N = 98). An analysis of the number of jobs held by employees reveals an interesting finding: a significant number of employees work two jobs (24%; N = 108) or more than two jobs (16%; N = 72), while the majority work only one job (60%; N = 269).

## 4. Results

**Fig 2:**
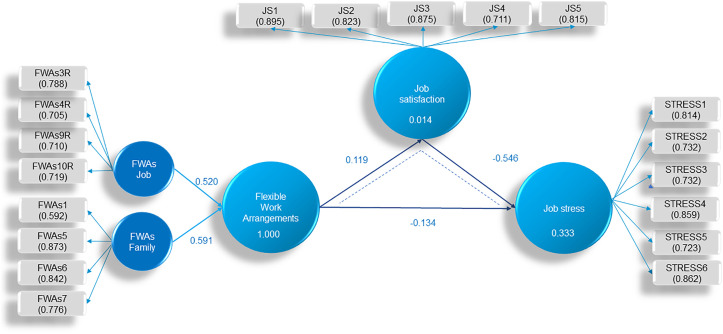
An estimate of the path coefficient for the indirect relationships between Flexible work arrangements and Job stress through Job satisfaction. Source: The authors’ research.

The research on the direct effects of Flexible work arrangements on Job satisfaction and Job stress, the direct effects of Job satisfaction on Job stress, as well as the mediating role of Job satisfaction in the relationship between Flexible work arrangements and Job stress, was conducted using PLS-SEM analysis. The model includes a second-order formative construct, Flexible work arrangements, composed of two first-order reflective constructs (FWAs – Job and FWAs - Family). Job satisfaction and Job stress are first-order reflective constructs (reflected through indicators). These relationships are depicted in Fig 2. which also illustrates the values of the outer loadings.

The authors first conducted a reliability analysis of the measurement model and then tested the structural model. The reliability analysis of the measurement model involves the evaluation of formative and reflective constructs, i.e., the analysis of the outer model. The formative construct in the model was assessed by analyzing the outer weights of the indicators, as well as their significance, using Standard Deviation, T-statistics, p-values, and the Variance Inflation Factor (VIF), see Table 2.

**Table 2:** Analysis of formative constructs in the outer model and values of variance inflation factor.

Relationship	Outer weights	St. Dev.	T Stat.	*p* values
**FWAs Family ->** **Flexible work arrangements**	0.591	0.024	24.686	0.000
**FWAs Job ->** **Flexible work arrangements**	0.520	0.023	22.857	0.000
**Variance inflation factor – VIF of formative construct**				
**FWAs Family**	1.62		Criterion < 3.3 [76]	
**FWAs Job**	1.62			

Source: The authors’ research.

Based on the analysis of the formative construct in the outer model and the presented Variance Inflation Factor (VIF) values, it can be concluded that both first-order constructs (FWAs – Family and FWAs – Job) have statistically significant relationships with the second-order formative construct FWAs, as the p-value is < 0.05, and the VIF values for the formative construct are < 3.3. Reflective indicators were analyzed to test the reflective constructs in the model, along with internal consistency reliability, convergent validity, and discriminant validity. The results are presented in Table 3.

**Table 3:** Outer loadings, assessment of measurement model reliability (Internal Consistency and Convergent Validity), and VIF.

Item	FWAs Family	FWAs Job	Job satisfaction	Job stress	Cronbach’s α	Composite Reliability	AVE	VIF
**FWAs1**	0.592				0.775	0.858	0.606	1.177
**FWAs5**	0.873							3.19
**FWAs6**	0.842							3.097
**FWAs7**	0.776							1.449
**FWAs10R**		0.719			0.711	0.821	0.535	1.383
**FWAs3R**		0.788						1.441
**FWAs4R**		0.705						1.335
**FWAs9R**		0.710						1.384
**JS1**			0.895		0.885	0.915	0.683	3.165
**JS2**			0.823					2.385
**JS3**			0.875					2.846
**JS4**			0.711					1.645
**JS5**			0.815					1.737
**STRESS1**				0.814	0.879	0.908	0.623	2.241
**STRESS2**				0.732				1.621
**STRESS3**				0.732				2.085
**STRESS4**				0.859				2.384
**STRESS5**				0.723				2.082
**STRESS6**				0.862				2.664

Source: The authors’ research.

Outer loadings above 0.708 should be retained, as they indicate that the construct explains more than 50% of the variance in the indicators, ensuring acceptable indicator reliability [77,78]. Instead of automatically eliminating indicators with loadings <0.70, the effects of their removal on other reliability and validity measures should be carefully examined. Indicators with loadings between 0.40 and 0.708 should be removed only if their deletion leads to an increase in internal consistency reliability or convergent validity above the proposed threshold. Therefore, indicators with lower loadings should sometimes be retained. According to the authors Hair, Ringle and Sarstedt [79] indicators with very low loadings (<0.40) should always be eliminated from the measurement model. Based on these criteria for indicator loadings, the items FWAs2R, FWAs8R, and FWAs11 had to be removed from further analysis due to their loadings being below the acceptable level. Cronbach’s α represents a measure of internal consistency to determine how closely related the items within a construct are. The values in the table range from 0.711 (FWAs – Job) to 0.885 (Job satisfaction). Dakduk, González and Portalanza [80] (p. 7) emphasize that values below 0.6 are unacceptable, values between 0.6 and 0.7 are minimally acceptable, values between 0.7 and 0.8 are acceptable, and values between 0.8 and 0.9 are considered very good. Composite Reliability assesses how consistent and reliable the items within a scale are in measuring the construct. The values for composite reliability range from 0.821 (FWAs – Job) to 0.915 (Job satisfaction). Hair et al. [81] indicate that the lower threshold for acceptable composite reliability is 0.7. Average Variance Extracted (AVE) represents the proportion of variance in the indicators captured by the latent construct that they are intended to measure. Based on the values presented in the table, the AVE values range from 0.535 (FWAs - Job) to 0.683 (Job satisfaction). Dash and Paul [82] state that the lower limit for acceptable AVE is 0.5. The lower threshold for acceptable VIF values, according to Kock [76] is < 3.3, while Akinwande, Dikko and Samson [83] note that VIF values between 5 and 10 indicate high correlation, which may be problematic. Based on the presented values, it can be concluded that all criteria are met. After assessing the reliability of the measurement model, Table 4. refers to results of the strictest criterion, the Heterotrait-Monotrait (HTMT) ratio.

**Table 4:** Discriminant validity – results of HTMT criterion.

Variable name	FWAs Family	FWAs Job	Job satisfaction
**FWAs Job**	0.818		
**Job satisfaction**	0.143	0.139	
**Job stress**	0.181	0.308	0.575

Source: The authors’ research.

The Heterotrait-Monotrait (HTMT) ratio is a relatively newer approach and is considered a stricter test of discriminant validity compared to traditional methods such as the Fornell-Larcker criterion. Hair et al. [77] (p. 79) highlight that the threshold for acceptable HTMT values is 0.9, with values above 0.9 indicating issues with discriminant validity. Based on the obtained results, it can be concluded that the criterion is met. After confirming that the measurement model is reliable, the next step involves testing and analyzing the structural model. The results obtained from the bootstrapping analysis are presented in Table 5.

**Table 5:** Results of bootstrapping analysis.

Relationship	β	St. Dev.	T Statistics	*p* values	Hypothesis
**Flexible work arrangements ->** **Job stress**	−0.134	0.038	3.522	0.000	accepted
**Flexible work arrangements ->** **Job satisfaction**	0.119	0.052	2.286	0.022	accepted
**Job satisfaction ->** **Job stress**	−0.546	0.031	17.629	0.000	accepted
**Flexible work arrangements ->** **Job satisfaction ->** **Job stress**	−0.065	0.028	2.325	0.020	accepted

Source: The authors’ research.

**Fig 3:**
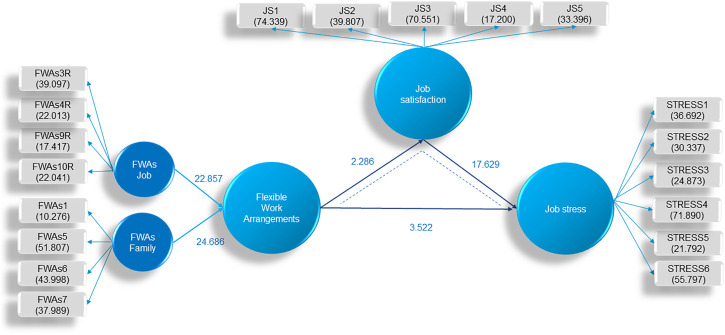
Path model representation with Bootstrapping analysis results. Source: The authors’ research.

Based on the results presented in Table 5. it was determined that there are statistically significant relationships between FWAs and Job stress (β = −0.134; t = 3.522; p = 0.000), FWAs and Job satisfaction (β = 0.119; t = 2.286; p = 0.022), Job satisfaction and Job stress (β = −0.546; t = 17.629; p = 0.000). The analysis revealed that partial mediation exists, as the indirect effect of FWAs on Job stress through Job satisfaction is significant (β = −0.065; t = 2.325; p = 0.020). These relationships are presented in Fig 3:

The R2 results indicate that changes in Job satisfaction are caused by 1.4% of FWAs, while the remaining 98.6% are due to other unexplored factors. Changes in Job stress are caused by 33.3% of FWAs, with the other 66.7% being attributed to unexplored factors.

According to the obtained results, it is necessary to understand the benefits that different forms of flexible work arrangements bring and have a positive effect on increasing the level of job satisfaction and reducing job stress. As already pointed out in the theoretical part, flexible work arrangements allow a greater level of autonomy for employees, and in addition contribute to the creation of a psychologically safer workplace, which reduces the level of anxiety, tension and chronic fatigue at work. Due to greater job satisfaction caused by greater autonomy of working hours and better work-life balance, the level of stress among employees has been further reduced, which has a positive impact on the mental health of employees.

The findings are particularly important in terms of the increased level of digitization, the implementation of remote work and greater new requirements for more efficient performance of work activities. It is necessary to further understand this relationship that allows organizations to develop a better strategy for managing human resources in the organization, reduce the level of intention to leave and retain talented employees, and ultimately all this affects greater overall competitiveness and business sustainability.

## Conclusions

The analysis of a sample of 448 highly educated young employees in Serbia, using the PLS-SEM method, showed that job satisfaction mediates the relationship between flexible work arrangements and job stress. Specifically, when employees can use flexible work arrangements, they demonstrate a higher level of job satisfaction, which directly contributes to reducing job stress. Additionally, flexible work arrangements have a direct positive effect on job satisfaction and a negative effect on stress levels, while job satisfaction further reduces job stress. The importance of job satisfaction and the implementation of flexible work arrangements in reducing job stress lies in their ability to influence the psychological well-being of employees directly. As a key factor, job satisfaction increases employee motivation and productivity and significantly reduces stress levels. When organizations enable flexible work arrangements, employees are provided with the opportunity to better balance their professional obligations with their personal lives, which leads to greater control over working hours and reduced pressure at work.

This way of doing business not only increases employee satisfaction, as evidenced in the works of authors [43,51,52,54], but also reduces job stress [27,47–50] which can lead to greater productivity and a reduction in employee turnover. The value of the company is also increased, as the FWAs in the form of hybrid workplace are proven to be positively correlated with the intangible assets [84] Organizations should adopt strategies that further enhance job satisfaction, such as professional development, mentoring, and recognition for achievements. Satisfied employees are less prone to stress and are more motivated to contribute to the organization. Trust between management and employees is crucial for the successful implementation of FWAs. Companies should encourage open communication and ensure employees have autonomy in planning their work [85].

Based on the overall research, some of the practical implications are as follows. In the IT sector, where deadlines are very short and expectations are high, the implementation of FWAs can significantly reduce the risk of burnout and increase productivity and employee performance. For example, Microsoft and Nordeus have introduced hybrid and remote work models, which reduced the turnover of young talented employees and additionally contributed to higher job satisfaction. Furthermore, the application of remote work and flexi-time has a positive effect on the psychological well-being of employees in the IT sector [86,87]. In the healthcare sector, which by its nature has certain specificities, the use of shift work contributes to better work-life balance (WLB). During the COVID-19 pandemic, hospitals in Serbia, as well as in the EU and the wider region, applied flexible work shifts and introduced psychological support programs for their staff, which led to reduced stress and burnout. This example provides a positive perspective for countries in transition economies [88]. In the education sector, employees engaged in teaching generally face high levels of job stress due to work overload. Accordingly, universities in Serbia adopted hybrid and asynchronous teaching models, such as online consultations, which further enabled lecturers to establish better WLB since they did not have to be present at multiple locations [9]. The next example refers to the retail and hospitality sector, where the implementation of shift work and job rotation in line with employees’ needs can further improve WLB, thereby increasing job satisfaction and reducing work-related stress. In line with this, Lidl Serbia and major hotel organizations in Serbia and the region experimented with flexible work schedules with the aim of reducing employee fatigue and better responding to the needs of employees with family obligations [89].

The connection with the SET refers not to the fact that if the organization provides the possibility of applying various forms of flexible work arrangements to employees, they “reciprocate” for example through a higher level of efficiency, productivity, effectiveness, performance. It should be emphasized that if the employees record a higher level of employee performance, the organizational performance will be better, which leads to a greater competitive advantage in that area of business and a sense of leadership arises where you determine the rules of the game in that sector. Employees also show less tendency to conflicts, greater resistance to stress and greater emotional connection with work.

Employers should implement personalized FWAs according to the type of work and employee preferences. They should also increase the level of transparency and communication, invest in a higher level of job satisfaction through the implementation of various non-material rewards such as recognition, a higher level of autonomy at work and better psychological safety. It is also necessary to develop a leadership approach that is based on trust and support for employees. And another very important thing is that organizations regularly monitor the levels of stress and satisfaction using various questionnaires and individual conversations with the aim of responding in a timely manner and reducing negative consequences.

On the other hand, employees should be proactive in seeking flexibility, they should openly communicate with their management about the need for such a way of working. It is essential that they manage their time effectively by setting clear boundaries between work and personal life. They should use flexibility for personal and professional development (in addition, they should engage in learning healthy routines and maintaining mental health). They should build trust with their superiors through appropriate work and efficient fulfilment of duties that are defined when they are not physically present in the office.

Recommendations for future research are directed towards studying various industries and cultures. This type of comparative study could reveal whether specific industries or cultural factors influence the effectiveness of FWAs in reducing stress through increased job satisfaction. It is necessary to conduct long-term research, specifically longitudinal studies, which could help identify lasting benefits or potential challenges that arise after a longer period of implementing these arrangements. Future research could examine how different types of FWAs uniquely impact job satisfaction and stress. Including perspectives from fields such as psychology, management, social policy, and economics can enrich the understanding of the mediating role of job satisfaction.

The primary limitations of the research are the number of respondents, the restriction to a single country, the duration of the study (longer-term research could yield different findings), etc.

## Supporting information

S1 File Data PLOS ONE FWAS 448. (XLSX)
